# Monoclonal Antibodies 13A4 and AC133 Do Not Recognize the Canine Ortholog of Mouse and Human Stem Cell Antigen Prominin-1 (CD133)

**DOI:** 10.1371/journal.pone.0164079

**Published:** 2016-10-04

**Authors:** Kristina Thamm, Sylvi Graupner, Carsten Werner, Wieland B. Huttner, Denis Corbeil

**Affiliations:** 1 Tissue Engineering Laboratories, Biotechnology Center (BIOTEC), Technische Universität Dresden, Dresden, Germany; 2 DFG-Research Center and Cluster of Excellence for Regenerative Therapies Dresden (CRTD), Technische Universität Dresden, Dresden, Germany; 3 Institute for Biofunctional Polymer Materials, Leibniz Institute of Polymer Research Dresden, Dresden, Germany; 4 Max Planck Institute of Molecular Cell Biology and Genetics, Dresden, Germany; University of British Columbia, CANADA

## Abstract

The pentaspan membrane glycoprotein prominin-1 (CD133) is widely used in medicine as a cell surface marker of stem and cancer stem cells. It has opened new avenues in stem cell-based regenerative therapy and oncology. This molecule is largely used with human samples or the mouse model, and consequently most biological tools including antibodies are directed against human and murine prominin-1. Although the general structure of prominin-1 including its membrane topology is conserved throughout the animal kingdom, its primary sequence is poorly conserved. Thus, it is unclear if anti-human and -mouse prominin-1 antibodies cross-react with their orthologs in other species, especially dog. Answering this issue is imperative in light of the growing number of studies using canine prominin-1 as an antigenic marker. Here, we address this issue by cloning the canine prominin-1 and use its overexpression as a green fluorescent protein fusion protein in Madin-Darby canine kidney cells to determine its immunoreactivity with antibodies against human or mouse prominin-1. We used immunocytochemistry, flow cytometry and immunoblotting techniques and surprisingly found no cross-species immunoreactivity. These results raise some caution in data interpretation when anti-prominin-1 antibodies are used in interspecies studies.

## Introduction

For more than a decade, prominin-1 (alias CD133) has emerged as a useful cell surface antigen of neural progenitors and hematopoietic stem cells allowing their immunoisolation based on specific monoclonal antibodies (mAbs) (reviewed in Refs [1–3]). Prominin-1 also highlights putative progenitors or stem cells in other somatic tissues notably prostate, kidney, liver and skin [4–7].

The expression of prominin-1 is not restricted to stem cells given that numerous differentiated epithelial cells and non-epithelia cells, particularly photoreceptors and glial cells, express it [8, 9]. Prominin-1 can also be found at the apical plasma membrane of epithelial cells present in the kidney and mammary glands among others ([10–12]; reviewed in Refs [1, 13]). In polarized epithelial cells, prominin-1 is concentrated in microvilli and primary cilia [12, 14].

Its detection in the ductal epithelia of glandular organs such as the pancreas, liver and salivary glands is important because they host cells with dedifferentiation capacities [11]. This suggests that prominin-1 marks facultative stem cells, which might be activated during regeneration [15]. The detection of prominin-1 in human cancer-initiating cells from various organs brought an international interest to this molecule as a specific biomarker of cells with stem cell properties, and, excitingly, as a potential target for cancer eradication [13, 16–19].

**Fig 1:**
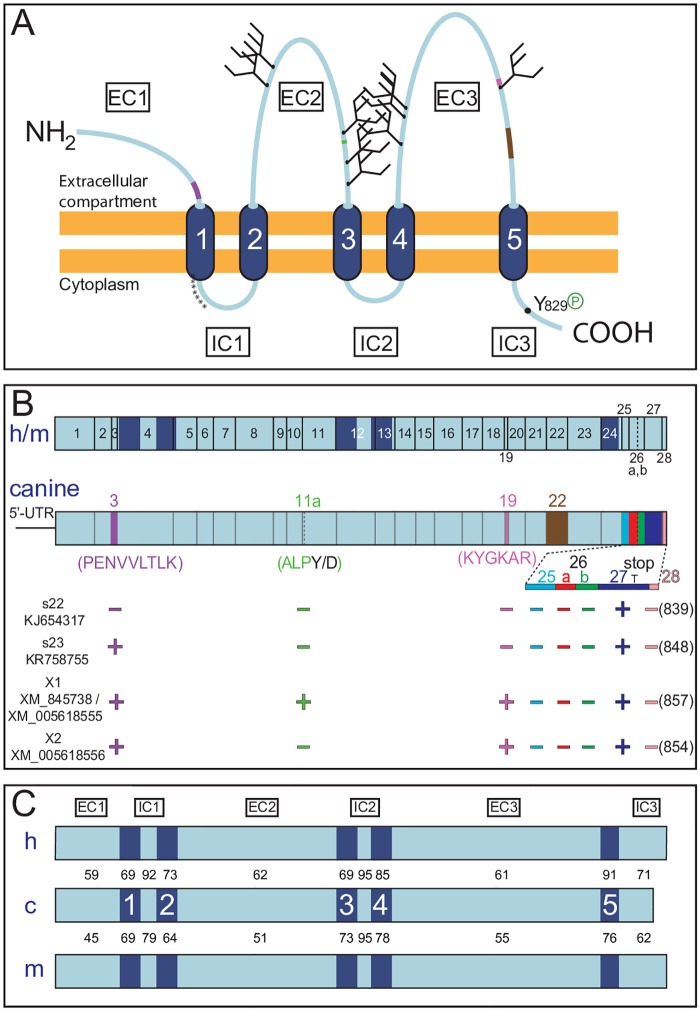
Comparison of canine prominin-1 with its human and mouse orthologs. (**A**) Membrane topology. Canine prominin-1 contains an extracellular N-terminal domain (EC1), five transmembrane segments (1–5) separating two small intracellular (IC1 and IC2), two large extracellular (EC2 and EC3) loops, and an intracellular C-terminal domain (IC3). The latter harbors a phosphorylation (P) site at tyrosine residue 829 for Src and Fyn tyrosine kinases. The asterisk stretch indicates a cysteine-rich region at the transition of the first transmembrane segment and IC1 domain. The EC2 and EC3 domains contain nine potential N-glycosylation sites (forks). The positions of the alternative exons 3 (purple), 11a (green), 19 (pink) and 22 (brown) found in the extracellular domains are indicated. (**B**) Genomic organization. The organization of human (h) and mouse (m) *PROM1* genes is shown and compared to the canine equivalent (top panel). Vertical lines indicate exon boundaries and dark blue zones highlight the position of the transmembrane segments. Exon numbering begins with the one bearing the start codon. For simplicity, the details of the 5’-untranslated region (UTR) are not shown (see S1B Fig). Facultative exons included in the coding sequences are depicted in different colors and amino acid sequences of exons 3, 11a, 19 and 22 are indicated in parentheses (see also S2A Fig). The new canine prominin-1 splice variants (s22, s23) and two additional predicted ones (*X1*, *X2*) including their GenBank accession numbers are presented (bottom panel). The presence (+) or absence (−) of an exon and the consequential protein sequence length (number of amino acids) are indicated. T represents the stop codon within exon 27. (**C**) Amino acid identity. The percentage of amino acid identity of each individual structural domain of canine (c) prominin-1 with its corresponding counterparts in human and mouse orthologs is presented.

Prominin-1 belongs to a family of cholesterol-binding pentaspan membrane glycoproteins expressed throughout the animal kingdom [20] (Fig 1A). In mammals, two *PROM* genes are described, and three distinct ones are found in non-mammalian species [1]. Numerous splice variants of prominin-1 were identified in various species [1, 21, 22]. The genomic structure of both mammalian prominin paralogs is strikingly similar (introns are concordant in position and phase) and remarkably conserved across species [20, 23]. Despite this genomic feature, the amino acid sequence is poorly conserved among *PROM1* gene products. For instance, primate and rodent prominin-1 exhibit an average identity of 60%; this number drops to about 45% in fish, amphibians and birds [22–26]. In invertebrates (e.g., worm and fly) less than 25% of the mammalian residues are conserved [27, 28]. The low level of amino acid conservation of prominin-1 between species can explain the lack of cross-species immunoreactivity of specific prominin-1 antibodies between human and mouse proteins.

The physiological role of prominin-1 remains elusive. Most laboratories focus on its use as a cell surface marker. Only a few of them are actually investigating its function. Observations regarding the molecular and cellular biology of prominin-1 including mutations in the human *PROM1* gene and data obtained from its knockout in murine models have led to its putative role as a scaffolding protein involved in the organization of plasma membrane protrusions. For example, prominin-1 localizes in membrane evaginations at the base of the photoreceptor outer segment [8, 29–31]. A lack of prominin-1 causes a defect in outer segment morphogenesis that can lead to blindness (reviewed in Refs [32, 33]).

Until now, most studies describing the expression of mammalian prominin-1 were limited to human and murine tissues. The dog is an interesting alternative as a large animal model, particularly to study stem cell-based regeneration and cancers [34, 35]. Indeed, close similarities to the human physiology and anatomy make the dog an attractive model to study various organ systems. Moreover, dogs develop cancers that share numerous features with human malignancies [36]. In the absence of specific antibodies against canine prominin-1, studies investigating its expression in dog tissues or cells derived therefrom rely on antibodies directed against its human and murine orthologs (see a non-exhaustive list in Table 1). For instance, mAbs 13A4 and AC133 that were used to identify and clone murine and human prominin-1, respectively [12, 37], are utilized to characterize prominin-1–expressing canine cells (Table 1).

**Table 1:** Studies on canine tissues and/or cell lines using anti-prominin-1 antibodies.

Study/year	Ref.	Canine tissues or cell lines investigated	Application*	Antibody (name/clone§) applied	Host/clonality^¶^	Immuno-gen‡
Lamerato-Kozicki *et al*, 2006	[38]	Hemangiosarcoma cell lines Blood samples	FC IC**	13A4^1^	Rat/ma	m
Wu *et al*, 2006	[39]	Bone marrow-derived endothelial progenitors	IC FC	hCD133^2^ hCD133^3^	Mouse/ma	h
Valenzuela *et al*, 2008	[40]	Skin-derived neuroprecursor cells from biopsies	IC	unspecified^4^	Rabbit/pa	h
Xia *et al*, 2009	[41]	Circulating endothelial progenitors	FC	AC133^2^ (CD133/1)	Mouse/ma	h
Cocola *et al*, 2009	[42]	Primary mammary cells from spontaneous breast cancers	WB	K-18^6^	Goat/pa	h
Stoica *et al*, 2009	[43]	Glioblastoma tissues Primary tumor cells	FC IHC IC	13A4^1^	Rat/ma	m
Turner *et al*, 2010	[44]	Remodeled small intestinal submucosa-extracellular matrix implants	IHC	unspecified^4^	Rabbit/pa	h
Cogliati *et al*, 2010	[45]	Hepatocellular carcinoma Cholangiocellular carcinoma	IHC	ab19898^4^	Rabbit/pa	h
Ito *et al*, 2011	[46]	Lymphoid progenitor cells from B-cell lymphoma biopsies	FC	13A4^1^	Rat/ma	m
Blacking *et al*, 2012	[47]	Various cancer cell lines Primary osteosarcoma	FC	13A4^1^	Rat/ma	m
Walton *et al*, 2013	[48]	Primary neural stem and progenitor cells Brain tissues	IHC	AC133^2^ (CD133/1) 293C3^2^ (CD133/2)	Mouse/ma	h
Moulay *et al*, 2013	[49]	Prostate carcinoma cell lines Prostate cyst-derived cell lines	FC	13A4^1^ 315-2C11^5^	Rat/ma	m
Fujimoto *et al*, 2013	[50]	Hepatocellular carcinoma cell lines	FC	13A4^1^	Rat/ma	m
Guth *et al*, 2014	[51]	Tumor biopsies from melanoma and osteosarcoma Osteosarcoma cell lines Melanoma cell lines	FC	AC133^2^ (CD133/1)	Mouse/ma	h
Gorden *et al*, 2014	[52]	Hemangiosarcoma cell lines	FC	AC133^2^ (CD133/1)	Mouse/ma	h
Tanabe *et al*, 2014	[53]	Lung adenocarcinoma cell line	WB	13A4^1^	Rat/ma	m
Michishita *et al*, 2014	[54]	Hepatocellular carcinoma cell line	FC	13A4^1^	Rat/ma	m
Liu *et al*, 2015	[55]	Prostate adenocarcinoma cell line ct1258	FC	13A4^1^	Rat/ma	m
Suzuki *et al*, 2015	[56]	Lingual granular cell tumors	IHC	ab19898^4^	Rabbit/pa	h
Fernandez *et al*, 2016	[57]	Glial tumors Kidney tissues	IHC	ab19898^4^	Rabbit/pa	h

* FC, flow cytometry; IC, immunocytochemistry; IHC, immunohistochemistry; and WB, western blot.

^§^ As stated by the seller;

^1^, eBioscience;

^2^, Miltenyi Biotec;

^3^, R&D Systems;

^4^, Abcam;

^5^, BioLegend; and

^6^, Santa Cruz Biotechnology.

^¶^ pa, polyclonal antibody; ma, monoclonal antibody.

^‡^ m, mouse; h, human.

** Data not shown.

Do antibodies generated against murine and human prominin-1 recognize the canine protein? This question is of interest not only because the amino acid identity between mammalian prominin-1 orthologs is modest, but also because discordant immuno-expression patterns of anti-human prominin-1 antibodies suggest that prominin-1 immunohistochemistry data should be interpreted with caution [11, 58, 59]. Furthermore, cross-reactivity of an anti-human prominin-1 antibody (i.e. AC141 mAb) with cytokeratin 18 was reported [60]. To address this question, we cloned prominin-1 cDNA from a beagle dog and the Madin-Darby canine kidney (MDCK) cell line and ectopically expressed it in the latter as an engineered green fluorescent protein (GFP) fusion protein. This allowed us to monitor its immunodetection using commercially available and homemade antibodies. To our surprise, we did not observe any cross-species immunoreactivity of the antibodies against human or mouse prominin-1 with the canine ortholog.

## Results

We cloned the entire open reading frame (ORF) of the *canis lupus familiaris* prominin-1 and overexpressed it as an engineered GFP-fusion protein in MDCK cells. This allowed us to evaluate the potential cross-species reactivity of available anti-prominin-1 mAbs directed against the human or mouse protein with their cognate in dog. The MDCK cell line was derived from kidney tissues of an adult female cocker spaniel in 1958 [61].

### Identification and Molecular Cloning of Canine Prominin-1 Splice Variants

Following *in silico* analysis and molecular cloning approaches (for the cloning strategy see Materials and Methods and S1A Fig), we identified and amplified distinct splice variants of canine prominin-1 from cDNA pools originating either from kidneys of adult beagles or MDCK cells (Fig 1 and S1 Fig; GenBank accession numbers KJ654317 and KR758755). The kidney was chosen as a mRNA source because prominin-1 has strong renal expression in rodents and humans [10, 12]. Predicted prominin-1 transcripts were also found in the NCBI database (GenBank entries: XM_845738, XM_005618555 and XM_005618556). Comparing these sequences with the corresponding *prom1* gene sequence located on chromosome 3 (NW_003726054.1) using the Basic Local Alignment Search Tool (BLAST) program showed that introns are conserved in position and phase with human and mouse *prom1* genes (Fig 1B and S1 Table). All exon/intron boundaries in the canine ortholog conform to the GT-AG rule (S1 Table).

The ORF showed that symmetric exon 3 –which encodes the 9-amino acid stretch PENVVLTLK in the extracellular N-terminal domain of prominin-1 –is alternatively spliced in the dog as previously reported for prominin-1 splice variants s1 and s2 in human and mouse [62, 63] (Fig 1A and 1B). We also highlighted one cDNA clone that lacks another exon (exon 22) in the third extracellular domain (Fig 1A and S2A Fig). Two other exons (11a and 19) are located in the second and third extracellular domains, respectively, and are predicted to be facultative (Fig 1B and S1 Table). Interestingly, the mini-exon 11a (residues ALP), which is located between exons 10 and 11 according to human (or mouse) gene structure, is also reported for amphibians and fish, but not yet in mammals [22, 25]. Strikingly, exon 27 in the dog harbors a stop codon generating a truncated cytoplasmic C-terminus that has not been found in any other species until now (Fig 1B and S2B Fig). This exon contains the entire 3’-untranslated region (UTR) and a polyadenylation consensus sequence (AAUAAA) 12 nucleotides upstream of a poly (A) tract (S2B Fig). Interestingly, analysis of the *prom1* gene revealed the presence of a facultative exon 26a (SSVLGTWHFTL) between exons 24 and 27 (S2B Fig), which was found in mouse prominin-1.s3 to s6 variant and is predicted for human and rat (S2C Fig) [21]. The use of this exon creates a distinct cytoplasmic C-terminal end. Exons 25, 26b and 28 reported in mouse and/or human *prom1* genes have not yet been identified in dog (Fig 1B and S1 Table).

Besides the ORF and in agreement with the existence of various 5’-UTR isoforms of human prominin-1 [64, 65], we amplified two alternative exons within the 5’-UTR (named A and B); a third one (C) is predicted (S1B Fig). The expression of different 5’UTRs in humans is tissue-specific and can vary with certain diseases (e.g., gliomas, colon cancers) (reviewed in Ref. [66]). In sum, these data suggest complex gene regulation of canine prominin-1 as reported for its counterparts in humans and mice. The functional significance of the alternatively spliced variants is unknown, but it indirectly suggests various interactions with other proteins—particularly by its alternative cytoplasmic C-terminal domain.

Following the nomenclature of prominin-1 splice variants [22, 23]–and with regard to the absence or presence of exon 3 –we named the new canine prominin-1 splice variants s22 and s23, respectively. They encode for 839 and 848 amino-acid proteins, respectively (Fig 1B).

### Modest Amino Acid Conservation of Mammalian Prominin-1

**Fig 2:**
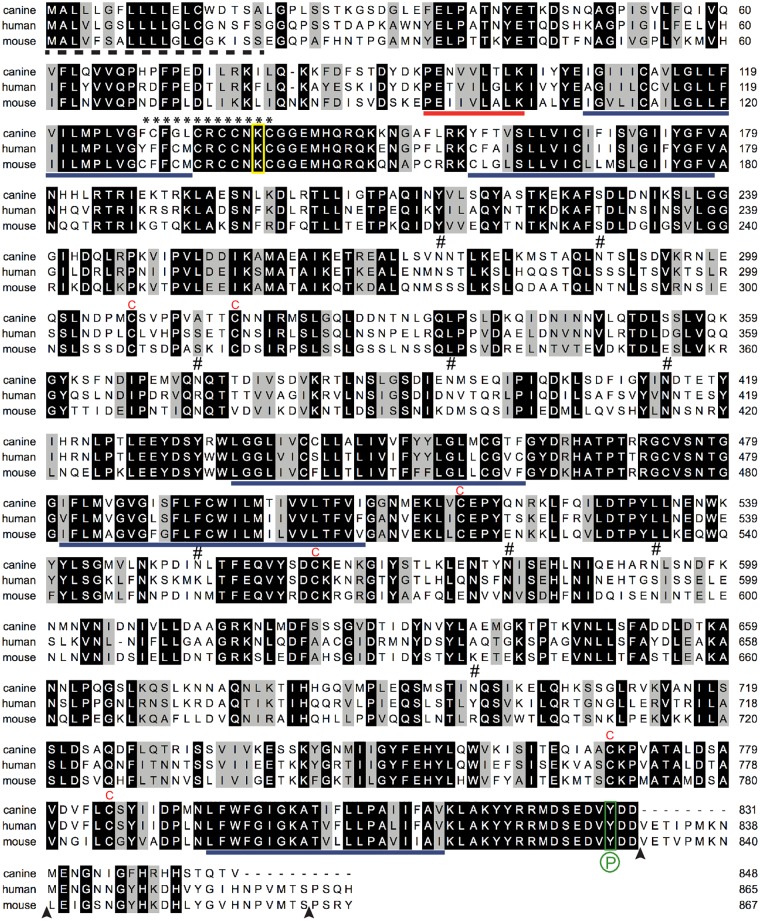
Comparison of the amino acid sequence of canine, human and mouse prominin-1. The canine prominin-1.s23 sequence determined in this study (GenBank Accession No. KR758755; top) was aligned with human (AF027208; middle) and mouse prominin-1.s2 (NM_001163577; bottom). Black and grey backgrounds indicate identical and similar amino acid residues, respectively; dashed line, putative signal peptide; solid blue lines, predicted transmembrane segments; solid red line, exon 3; asterisk stretch, cysteine-rich region; red C, conserved cysteine residue in extracellular domains; #, potential N-glycosylation site in canine prominin-1; yellow box, conserved lysine potentially involved in the interaction with HDAC6; green box, conserved Src/Fyn phosphorylation (P) site; and arrowhead, exon boundaries.

A multiple sequence analysis of canine prominin-1 with human, mouse and rat orthologs revealed that the common characteristics of this pentaspan membrane glycoprotein are conserved (Fig 2, data not shown) [24]. For instance, six cysteine residues in the first and second extracellular loop are likely to form disulfide bridges and are found in all species (Fig 2, letter C in red). The unusual extra cysteine (residue 624) in human prominin-1 is absent in the canine sequence like in mouse and rat.

The cysteine-rich sequence at the transition of the first transmembrane domain and the first cytoplasmic loop is also present in canine prominin-1 (Fig 2, asterisks). The lysine 138 within the cysteine-rich domain, which is implicated in the interaction with histone deacetylase 6 (HDAC6), is conserved (Fig 2, yellow box) [67]. This holds true for the tyrosine 829 that can be phosphorylated by Src and Fyn tyrosine kinases in human prominin-1 (Figs 1A and 2, green box, P) [68]. The latter posttranslational modification can promote the interaction of prominin-1 with the regulatory subunit (p85) of phosphoinositide 3-kinase (PI3K) leading to the activation of PI3K/Akt pathway [69]. Nine N-glycosylation sites are found in the extracellular domains of canine prominin-1. Only two of them are conserved in position with human and mouse proteins (Fig 2, symbol #).

The pairwise sequence comparison revealed that canine prominin-1 exhibits an overall amino acid identity of 66% and 57% with its human and mouse orthologs, respectively, while the human and mouse sequences show 61% identity. The similarity of canine prominin-1 reaches 74% and 66% with human and mouse proteins, respectively. Although the two small intracytoplasmic loops display a significant amino acid identity between mammalian prominin-1 (79 to 95%), the three large extracellular domains are modestly conserved (Fig 1C). Only 60% of the residues exposed to the extracellular milieu are conserved between the canine and human proteins, and this number decreases to 45–55% in the murine sequence. The cytoplasmic C-terminal domain of canine prominin-1 is significantly different between human and mouse due to the presence of a stop codon in exon 27 (Figs 1B and 2).

Given the limited amino acid conservation between the canine prominin-1 and various mammalian orthologs, the cross-species reactivity of antibodies directed against human or mouse prominin-1 with the canine protein is questionable—particularly when they are directed against the large extracellular loops or the cytoplasmic C-terminal end.

### Canine Prominin-1 Is Targeted to the Apical Plasma Membrane of Polarized Epithelial Cells

To further characterize the canine prominin-1, we ectopically expressed it as a GFP-fusion protein in MDCK cells. We expressed the canine prominin-1.s23 variant because it has the same extracellular domains as the human prominin-1, which is recognized by commercial and homemade mAbs (see below). The GFP allows us to monitor its expression in the absence of specific antibodies. We have previously demonstrated that the addition of GFP in-frame with the cytoplasmic C-terminal domain of human or mouse prominin-1 did not interfere with their membrane topology nor their proper intracellular trafficking and targeting to a specific subdomain of polarized cells (see also below) [14, 70].

**Fig 3:**
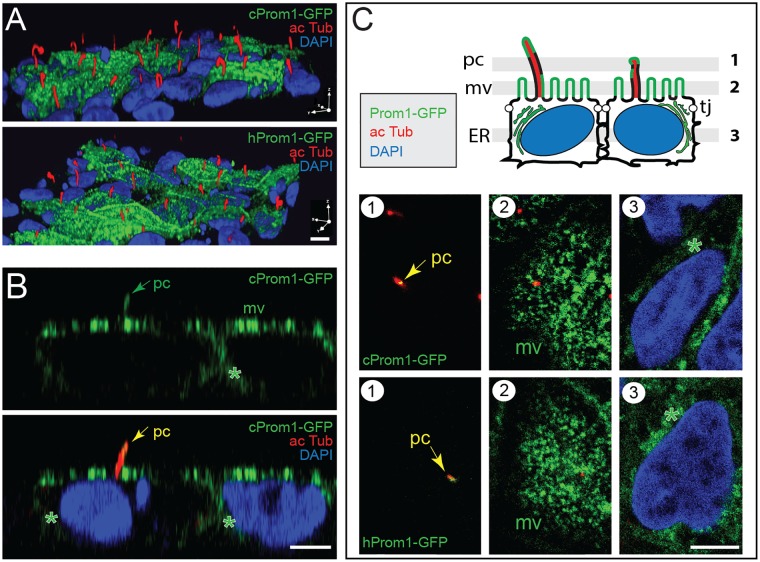
Canine prominin-1-GFP is found in microvilli and the primary cilium at the apical domain of polarized epithelial cells. (**A-C**) Stably transfected MDCK cells expressing either canine (c) or human (h) prominin-1-GFP (Prom1-GFP, green) were immunolabeled for acetylated α-tubulin (ac Tub, red) and their nuclei were counterstained with DAPI (blue). 3-dimensional reconstruction of 32 optical x-y sections (0.6 μm-slices) (A) and a single x-z section (B) are shown. The three single x-y sections (1–3) displayed in (C) are located as illustrated in the cartoon above. They reveal the presence of prominin-1-GFP at the apical plasma membrane with fluorescent signals characteristic of microvilli (mv) and the primary cilium (pc, arrow; see also S1 Video) as well as in intracellular structures such as the endoplasmic reticulum (ER, asterisk). Tj, tight junction. Scale bars, 5 μm.

To determine the distribution of canine prominin-1-GFP in polarized epithelial cells, we grew MDCK cells for 6 days post-confluence. Confocal laser scanning microscopy analysis revealed the presence of the fusion proteins at the apical domain of cells with a punctate staining pattern typical for microvilli (Fig 3A–3C mv). The recombinant protein was also found in primary cilia, which are highlighted by acetylated tubulin labeling (Fig 3A–3C, pc; S1 Video). The single x-z (Fig 3B) and x-y sections (Fig 3C) indicate a fluorescent signal in cytoplasmic vesicles consistent with the intracellular transport of prominin-1-GFP en route to the apical plasma membrane (asterisks). No significant GFP-fluorescence was observed at the basolateral domain (Fig 3B and 3C, S1 Video). The same distribution pattern of human prominin-1-GFP fusion protein was observed upon its overexpression in MDCK cells (Fig 3A and 3C). Thus, canine prominin-1 is targeted to the apical plasma membrane of polarized epithelial cells and concentrated in plasmalemmal protrusions found therein as previously reported for human and mouse prominin-1 [11, 12, 14, 71–73].

### Commercial Monoclonal Antibodies Do Not Recognize Canine Prominin-1 by Immunocytochemistry and Flow Cytometry

To evaluate if commercial antibodies directed against human and mouse prominin-1 can cross-react with the canine protein as assumed in several studies (Table 1), we established stably transfected MDCK cells expressing human and mouse prominin-1 as positive controls, in addition to canine prominin-1-GFP^+^ cells. We chose splice variants that are recognized by mAbs directed against human (i.e. clones AC133, AC141 and 293C3) and mouse (clone 13A4) prominin-1 [12, 37]. The CD133/1 (recognized by AC133 antibody) and CD133/2 (recognized by AC141 and 293C3 antibodies) epitopes are found in the second extracellular loop of human prominin-1 [74, 75], while the same domain in mouse prominin-1 contains the 13A4 epitope [8]. The structural domains of both extracellular loops, in terms of exons, are conserved within the dog prominin-1.

**Fig 4:**
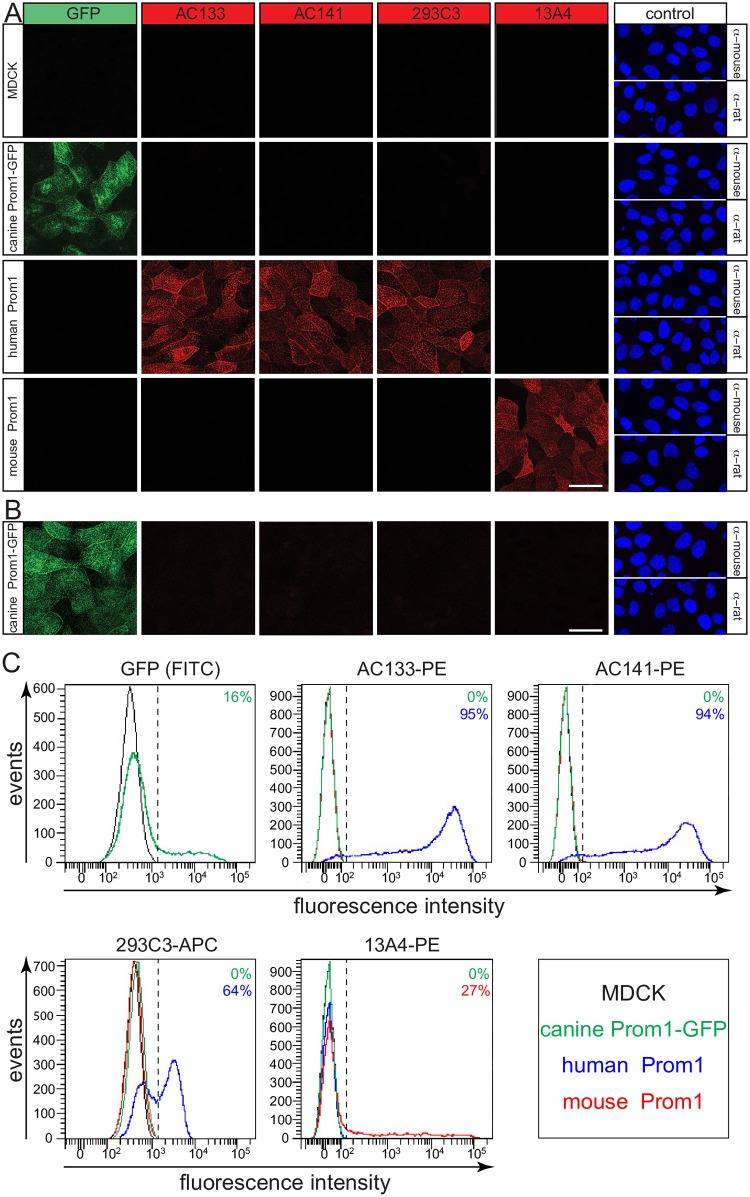
Commercially available anti-human and anti-mouse antibodies fail to detect canine prominin-1 by cytochemistry and flow cytometry. (**A-C**) The MDCK cells stably transfected with human or mouse prominin-1, canine prominin-1-GFP as well as wild type cells (MDCK) were analyzed either by immunocytochemistry (A, B) or flow cytometry (C) using mAbs AC133, AC141, 293C3 and 13A4. For immunocytochemistry, cells were either cell surface labeled in the cold (A) or permeabilized with saponin after fixation (B). As negative controls, only the secondary antibody was used, and cells were counterstained with DAPI (A, B). Scale bars, 30 μm. For flow cytometry, cells were directly incubated with fluorochrome (PE or APC)-coupled mAbs as indicated. Cells expressing prominin-1-GFP were examined using the FITC channel. The vertical dashed lines indicate the cut-off for cells positive for a given prominin-1. These were established using unstained cells or those labeled with the irrelevant anti-human CD34 antibody conjugated to the appropriate fluorochrome (S4 Fig). The color code indicates the corresponding cell line used, and the percentage of positive cells is specified in each panel.

The cell surface immunostaining of untransfected MDCK cells with the above antibodies revealed that none of them detected canine prominin-1 (Fig 4A). More strikingly, the overexpressed canine prominin-1-GFP was not recognized by any tested mAb (Fig 4A). In contrast, the antibodies could detect either human or mouse prominin-1 (Fig 4A). To rule out that canine prominin-1-GFP did not reach the cell surface but instead localized to vesicular structures underneath the plasma membrane, we repeated the staining upon cell permeabilization via a low concentration of saponin. Again, the anti-prominin-1 antibodies failed to detect the endogenous and ectopically expressed canine prominin-1 (Fig 4B).

We next wondered if the addition of GFP at the cytoplasmic C-terminal domain of canine prominin-1 somehow influences its tertiary structure—particularly the second extracellular loop—in a way that impedes the epitope accessibility. To answer this question, we repeated the immunocytochemistry using the human prominin-1-GFP fusion protein where the GFP is also added at the C-terminal end. In contrast to canine prominin-1-GFP, we could detect the fusion protein with all mAbs (i.e. AC133, AC141 and 293C3) directed against human prominin-1 (S3A Fig). This indicated that the protein tag did not influence the accessibility of the corresponding epitopes. Again, the mAb 13A4 did not recognize the human prominin-1 (S3A Fig). The lack of immunodetection of canine prominin-1 can also result from other molecular constrains—notably the incorporation of prominin-1 in ganglioside GM_1_-enriched membrane microdomains that occurs when epithelial cells are grown as a polarized monolayer [10, 73, 76] (reviewed in Ref. [77]). Thus, we re-analyzed MDCK cells with flow cytometry after substrate detachment. We used antibodies that were directly coupled to a fluorochrome. None of these antibodies cross-reacted with endogenous prominin-1 or canine prominin-1-GFP, although the anti-human and anti-mouse prominin-1 antibodies highlighted their respective antigen (Fig 4C). No positive cells were observed without primary antibody or with an irrelevant antibody, i.e. anti-human CD34 antibody (S4 Fig).

### Commercial Monoclonal Antibodies Do Not Recognize the Canine Prominin-1 by Immunoblotting

**Fig 5:**
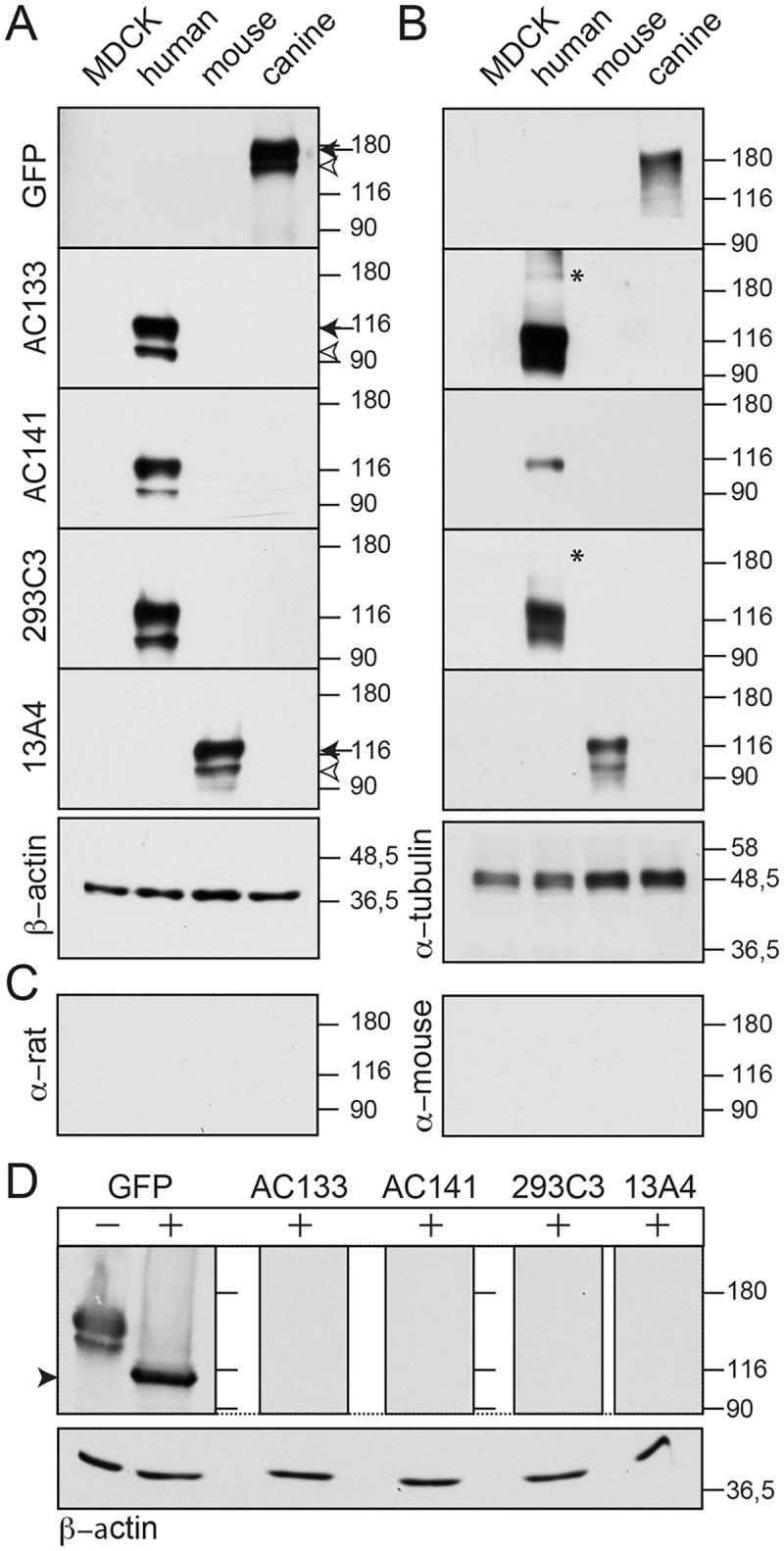
The commercial anti-human and anti-mouse antibodies fail to detect canine prominin-1 by immunoblotting. (**A-C**) Detergent lysates prepared from MDCK cells stably transfected with human or mouse prominin-1, canine prominin-1-GFP as well as wild type cells (MDCK) were analyzed by SDS-PAGE under reducing (A, C) and non-reducing (B) conditions and immunoblotting using mAbs AC133, AC141, 293C3, and 13A4 or polyclonal antibody against GFP. β-actin and α-tubulin were used as loading controls. As negative controls, only the secondary antibody (as indicated) was used (C). (**D**) Lysates from canine prominin-1-GFP transfected cells were incubated with (+) or without (–) PNGase F prior to immunoblotting with anti-GFP antibody or others (as indicated). The arrow and open arrowhead indicate the plasma membrane-associated form and endoplasmic reticulum-associated form of prominin-1, respectively. Asterisk indicates potential disulfide-bridged prominin-1 dimers or multimers, and the black arrowhead shows deglycosylated prominin-1. Molecular mass markers (kDa) are indicated. The original and uncropped blots are presented in S5 Fig.

We investigated whether commercial antibodies can detect canine prominin-1 by immunoblotting. Proteins in detergent lysates prepared from the established MDCK cell lines were separated by SDS-polyacrylamide-gel electrophoresis (SDS-PAGE) under reducing condition (i.e. in presence of β-mercaptoethanol) and analyzed by blotting. As expected from the immunocytochemistry and flow cytometry, none of the antibodies directed against human and mouse prominin-1 detected the endogenous prominin-1 of MDCK cells or its overexpressed GFP fusion protein (Fig 5A). The latter can be observed using an anti-GFP antibody and shows the expected higher molecular weight. The two-band pattern of recombinant prominin-1 (canine, human and mouse proteins) is consistent with its overexpression as demonstrated in earlier reports [12, 21, 71]. The immunoreactive band with an apparent molecular mass of ≈115–120 kDa (or ≈160–170 kDa in the case of GFP fusion protein) represents proteins at the plasma membrane (Fig 5A, arrow). The ≈100–105 kDa (≈150 kDa for prominin-1-GFP) forms are found in the endoplasmic reticulum and/or an early Golgi compartment (Fig 5A, arrowhead). The antibodies also failed to detect the canine prominin-1 under non-reducing conditions (Fig 5B). The mAbs AC133 and 293C3 detected a faint band at ≈190 kDa (Fig 5B, asterisk) suggesting the formation of dimers or multimers.

Given that we were monitoring the GFP-fusion protein for canine prominin-1, we also performed the immunoblotting of lysates from MDCK cells expressing human prominin-1-GFP. Again, anti-human prominin-1 antibodies recognized the human, but not the canine, fusion protein (S3B and S3C Fig). The expression levels of human and canine GFP-fusion proteins were comparable (S3D Fig).

Because of the debate about the role of glycosylation in the recognition of certain human prominin-1 epitopes, notably CD133/1 (reviewed in Ref. [78]), we decided to remove the N-glycans prior to immunoblotting. Detergent extracts from MDCK cells expressing canine prominin-1-GFP were treated with PNGase F and analyzed by SDS-PAGE under reducing conditions. Upon detection with an anti-GFP antibody, the recombinant fusion protein is glycosylated as observed by the shift of the molecular mass from ≈150/170 to 115 kDa (Fig 5D, black arrowhead). This is consistent with the presence of nine N-glycosylation sites (Fig 2). However, the removal of sugar moieties did not unmask any detectable epitope so again none of the anti-human or mouse prominin-1 antibodies could recognize the canine prominin-1 (Fig 5D).

### Homemade Antibodies Do Not Recognize the Canine Prominin-1

**Fig 6:**
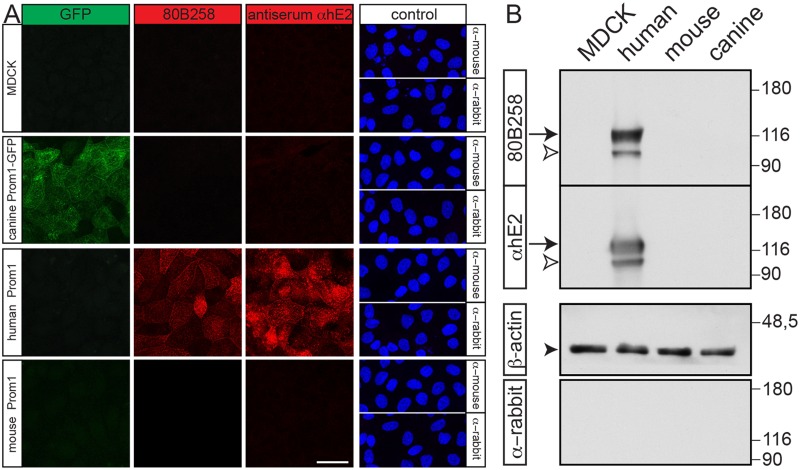
Homemade mouse monoclonal antibody 80B258 and rabbit antiserum αhE2 fail to detect canine prominin-1. (**A, B**) MDCK cells stably transfected with human or mouse prominin-1, canine prominin-1-GFP as well as wild type cells (MDCK) were analyzed either by immunocytochemistry (A) or immunoblotting under reducing conditions (B) using mouse mAb 80B258 or rabbit antiserum αhE2. As a negative control, only the secondary antibody (as indicated) was used (A, B). For immunocytochemistry, cells were counterstained with DAPI (A). For immunoblotting, β-actin was used as loading control (black arrowhead). The arrow indicates the plasma membrane-associated form of prominin-1, and the open arrowhead indicates its endoplasmic reticulum-associated form. The original and uncropped blots are presented in in S5 Fig. Scale bar, 30 μm.

Our laboratory has developed a mouse mAb and rabbit antiserum named 80B258 and αhE2, respectively, against human prominin-1 [10, 11]. They were generated using a fragment of its first extracellular loop corresponding to amino acid residues 240–388 of splice variant s2 as the antigen (Fig 2). This region exhibits 59% amino acid identity and 68% similarity between human and canine prominin-1. Only 49% amino acid identity is observed with the mouse ortholog. Both mAb 80B258 and antiserum αhE2 failed to recognize the endogenous prominin-1 as well as the ectopically expressed canine prominin-1-GFP in MDCK cells by immunocytochemistry and immunoblotting (Fig 6A and 6B, respectively). They recognized human prominin-1 or its GFP version, but not mouse prominin-1 (Fig 6 and S3B Fig), in agreement with a previous report [11].

## Discussion

Cells with self-renewal and differentiation properties like stem cells and cancer stem cells are described as the good and the bad players, respectively. The former are fundamental cellular components in the development and maintenance of tissue homeostasis with emerging utility in regenerative medicine. The latter appear to drive tumorigenesis and represent potential targets for new therapeutic approaches in cancer treatment. The clinical successes based on these cells rely on their proper identification, isolation and targeting. Thus, the discovery of improved cell surface biomarkers is critical.

One of the most promising is prominin-1 [79]. Its utility for the prospective identification and isolation of human and murine stem cells is now established, and specific tools such as antibodies are commercially available (see Table 1) [37, 80, 81]. The direct targeting of prominin-1 with biological drugs might become a useful approach to eradicate cancer stem cells ([82]; reviewed in Refs [83, 84]).

Nonetheless, the use of anti-human prominin-1 antibodies and the interpretation of data need to be considered with caution because discrepancies in immunohistochemical expression patterns of prominin-1 in human samples have been observed in stem cell niches and cancerous tissues [59]. Technically, the inconsistencies can be explained by a cross-reactivity of certain anti-human prominin-1 antibodies with other proteins [60] or by differences in the methods, e.g., tissue-fixation, antigen retrieval processes and antibody concentration [11, 59, 85, 86]. Biologically, the expression of different splice variants of prominin-1 carrying (or not) a given epitope or potential specific cleavage products may determine its immunodetection [23, 87].

The complexity of data analysis increases when the anti-prominin-1 antibodies are used in cross-species studies because prominin-1 proteins are poorly conserved among mammals. This is particularly true for the dog—a critical animal model for new cancer drugs and other applications. Dogs develop various cancers with strong similarities to human histology, biological behavior and response to conventional therapies. The rapid evolution of cancer in dogs allows timely assessment of novel therapies [36]. In this context, the use of prominin-1 in canine models is exponentially growing (Table 1). In the absence of specific anti-canine prominin-1 antibodies, investigators are using those against human and mouse proteins. This prompted us to evaluate their cross-reactivity with canine prominin-1. To achieve this goal, we cloned canine prominin-1 cDNA from two independent kidney mRNA sources—MDCK cells and adult beagle—and determined its genomic organization. A strong expression of prominin-1 in the kidney was observed in other mammals (e.g., mouse, rat and human) [10, 12, 24].

Interestingly, the genomic organization of the canine *prom1* gene is similar to human and mouse genes [8, 20]. This allows us to predict facultative and/or alternative exons as those identified in other species [21, 23]. We cloned and identified various prominin-1 splice variants harboring facultative exons (i.e. exons 3, 11a, 19, 22) in the extracellular domains of prominin-1 (Fig 1 and S2 Fig). It is important to bear in mind that prominin-1 is subjected to alternative splicing because different splice variants can carry specific epitopes. Their absence can impede the immunodetection of prominin-1. In mouse and human, the expression of distinct isoforms is developmentally regulated and tissue-specific, a situation that might occur in dogs as well [9, 21, 88].

Analysis of the canine *prom1* gene reveals an alternative exon (exon 26a) in the cytoplasmic C-terminal domain (S2 Fig). Its expression as demonstrated in murine glial cells [9] also hampers the detection of prominin-1 via antibodies directed against the amino acid residues of the alternative cytoplasmic C-terminal end (Table 1). The sequence analysis of this domain is particularly interesting because it differs significantly between canine and human/mouse/rat prominin-1 (Fig 2). Notably, exon 27 in canine prominin-1 harbors a stop codon that generates a truncated cytoplasmic C-terminus, which lacks homology with the last 15 amino acids of prominin-1 in other species. Antibodies generated against this part of human or mouse prominin-1 whether commercial (e.g., rabbit antiserum ab66141 (Abcam) and #PAB12663 (Abnova)) or homemade (αI3; [71]) will fail to detect the canine ortholog.

In addition to facultative exons that could interfere with canine prominin-1 immunodetection by certain antibodies, the amino acid sequence identity between canine and human/mouse prominin-1 is modestly conserved (Figs 1 and 2). This raises questions about its cross-species detection when using anti-human or anti-mouse prominin-1 antibodies. This issue was addressed here. We evaluated various anti-human prominin-1 antibodies including mouse mAbs AC133, AC141 and 293C3 that have been applied to canine samples in other studies (Table 1). AC133 recognizes the CD133/1 epitope in human prominin-1 while the other two antibodies mark the CD133/2 epitope.

Kemper and colleagues have proposed that both epitopes are located in the second extracellular loop of prominin-1 [74]. However, Zhao and coworkers suggested that mAb 293C3 can compete with a new antibody (CD133-scFv-1) that binds to the first extracellular loop of prominin-1 [75]. We also assessed two homemade antibodies (mouse mAb 80B258 and antiserum αhE2) generated against a segment of the first extracellular loop of human prominin-1 [10, 11]. The commercial rat mAb 13A4 that recognizes an epitope in the second half of the second extracellular loop of mouse prominin-1 was also tested [8].

For antigens, we used either the endogenous canine prominin-1 expressed by MDCK cells or a recombinant prominin-1, which we overexpressed in the same cell line as a GFP fusion protein. The added tag did not interfere with the intracellular transport of prominin-1-GFP and its targeting to the apical plasma membrane including the primary cilium (Fig 3, S1 Video) as demonstrated previously for murine and human prominin-1 [12, 14, 21, 71–73]. Here, we used a splice variant of canine prominin-1 that contains the same extracellular domains (i.e., corresponding exons) such as human and mouse prominin-1 that are recognized by their respective antibodies. Furthermore, human and mouse prominin-1 were overexpressed in MDCK cells and used as positive controls. The benefit of using the same cell line as a host is the ability to rule out any negative effect of the glycosylation status on the antibody recognition. This issue has catalyzed a large debate particularly with mAb AC133 [10, 72, 74, 78]. Moreover, the addition of a GFP tag did not interfere with the immunodetection of prominin-1 as shown for the human protein (S3 Fig). None of the antibodies mentioned above recognized native or recombinant canine prominin-1 using antibody-based techniques such as immunocytochemistry, flow cytometry and immunoblotting under reducing and non-reducing conditions. Likewise, anti-human antibodies did not detect mouse prominin-1 and vise versa. These results are in line with the low levels of amino acid conservation between the extracellular domains of prominin-1 orthologs.

Collectively, our data raise reservations about the prominin-1 detection in canine samples based on immunohistochemistry and flow cytometry using anti-human and anti-mouse prominin-1 antibodies. Considerations about the cross-species immunoreactivity should be noted and discussed when uncertainty of prominin-1 staining was observed [48]. To complement such an investigation, it might be appropriate to perform immunoblots to detect the proper immunoreactive prominin-1 bands. The tissue analysis of prominin-1 transcripts by *in situ* hybridization should also be considered [89, 90].

In conclusion, our experiments suggest that available antibodies against human and mouse prominin-1 do not cross-react with the canine ortholog, and hence some caution in data interpretation is necessary. Given the growing interest of prominin-1 as a biomarker of stem and cancer stem cells and dogs as experimental models, it is important to generate specific mAbs against canine prominin-1.

## Materials and Methods

### Molecular Cloning of Canine Prominin-1

The nucleotide and protein databases at the National Center for Biotechnology Information (NCBI) were searched by BLAST network services [91] using the human and mouse prominin-1 nucleotide sequences (GenBank accession numbers AF027208 and F026269, respectively) as probes. We obtained a predicted canine prominin-1 sequence (transcript variant *X1*, #XM_845738.4) derived from the whole genome shotgun sequence of chromosome 3 of *canis lupus familiaris* breed boxer (#NW_003726054.1). Based on this information, we designed various oligonucleotide primers (S2 Table, primer Nr. 1–5) to selectively amplify the canine prominin-1 by polymerase chain reaction (PCR). As a template, we prepared a cDNA pool from total RNA of MDCK cells (strain II, obtained from American Type Culture Collection, #ATCC^®^CCL-34^™^) using MultiScribe^™^ reverse transcriptase (#4368814, Applied Biosystems, Thermo Fisher Scientific). As a result, a 1.9-kb fragment of canine prominin-l was amplified (see S1A Fig). However, it did not contain the 5’- and the 3’-ends. The missing extremities were amplified by 5’- and 3’-rapid amplification of cDNA ends (RACE) using SMARTer^™^ RACE cDNA amplification kit according to the manufacturer’s instructions (#634858, Takara Bio Europe/Clontech, France). All resulting PCR DNA fragments were gel extracted (QIAquick Gel Extraction Kit, #28704, Qiagen GmbH, Helden, Germany) and subcloned into pCR^™^4-TOPO^®^ TA vector (Invitrogen). The identity of the inserts was determined by sequencing. This strategy identified three splice variants affecting the open reading frame of canine prominin-1 and two affecting its 5’UTR (S1 and S2 Figs).

The three overlapping cDNA fragments were combined using oligonucleotide primers carrying different specific restrictions sites (S2 Table, Nr. 8–13) for amplification. Briefly, the 5’-RACE cDNA fragment was subcloned into the pEGFP (enhanced green fluorescent protein)-N1 vector (Takara Bio Europe/Clontech) opened with XhoI and BamHI enzymes. The 1.9-kb fragment was ligated to the 5’-end after digestion with the SbfI and BamHI enzymes. The 3’-RACE cDNA fragment was then inserted in the same way using the introduced SacI and BamHI restriction sites. All restriction sites introduced for subcloning were removed using QuikChange^®^ II Site-Directed Mutagenesis Kit according to the manufacturer’s instructions (#200523, Agilent Technologies) with the oligonucleotide primers (Nr. 16–19) described in S2 Table. Note that the ORF of the prominin-1.s23 sequences are not in-frame with the GFP.

We also used PCR to obtain the canine prominin-1.s22 cDNA sequence from adult beagle dog kidney cells (dog kidney full length tissue cDNA, #DD-901, Zyagen, San Diego, CA) using oligonucleotide primers 6 and 7 (S2 Table). The resulting PCR DNA product was subcloned into pCR^™^4-TOPO^®^ TA vector.

All DNA fragments and the final sequences were verified by sequencing to confirm the absence of any reading mistake introduced by the DNA polymerase. The sequencing was performed at the Sequencing Facility of the Max Planck Institute of Molecular Cell Biology and Genetics (Dresden, Germany). The resulting sequences were deposited in GenBank database under the following accession number KJ654317.1 and KR758755.1; they are also presented in S1 and S2 Figs.

### Expression Plasmid Construction

The eukaryotic expression plasmid containing canine prominin-1.s23 sequence in-frame with GFP was generated by removing the terminal codon of prominin-1 as well as one adenine residue using QuikChange^®^ II Site-Directed Mutagenesis Kit with the oligonucleotide primers 14 and 15 (S2 Table). A linker of 11 amino acids is located between the prominin-1.s23 and the GFP sequence. The eukaryotic expression plasmids containing either mouse prominin-1.s1, human prominin-1.s2 or human prominin-1.s2-GFP have been described previously [12, 70, 72].

### Protein Sequence Analysis

The amino acid sequence deduced from canine prominin-1 cDNAs was compared with its mouse and human orthologs using CLUSTAL OMEGA [92, 93]. Pairwise sequence identity and similarity were analyzed using Sequence Identity And Similarity (SIAS) with BLOSUM62 matrix (gap open and gap extend penalties at 10 and 0.5, respectively) [94] on a Spanish server (http://imed.med.ucm.es/Tools/sias.html). The signal peptide and transmembrane domains were determined using the Consensus Constrained TOPology prediction web server (http://cctop.enzim.ttk.mta.hu) [95]. The Color Align Conservation program from Sequence Manipulation Suite (SMS; www.bioinformatics.org) was used to illustrate the output of the sequence alignment programs [96].

### Cell Culture and Transfection

MDCK cells (see above) were cultured in Dulbecco’s Modified Eagle Medium (DMEM, 1x, 1g/l D-Glucose, L-Glutamine, Pyruvate, GIBCO) containing 10% fetal calf serum (FCS, PAA, GE Healthcare), 100 U/ml penicillin and 100 μg/ml streptomycin (GIBCO), 10 mM HEPES and MEM Non Essential Amino Acids (1x, PAA) at 37°C in a humidified 5% CO_2_ atmosphere as described previously [71]. They were stably transfected with eukaryotic expression plasmids encoding either canine prominin-1.s23-GFP (see above), human prominin-1.s2, human prominin-1.s2-GFP [70, 72, 97] or mouse prominin-1.s1 [12] by electroporation using Amaxa^™^ Nucleofector^™^ Kit L according to the manufacturer’s instructions (#VCA-1005, Lonza, Cologne, Germany). Cells expressing the neomycin resistance gene were selected by introducing 500 μg/ml of geneticin (Thermo Fisher Scientific) into the culture medium and further expanded in a medium containing only 250 μg/ml of selective agent. To enhance the expression of the transgene, 10 mM sodium butyrate was added to the culture medium for 16 h prior to the analyses [71]. Under these conditions, 50–90% of neomycin-resistant cells expressed the recombinant prominin-1.

### Immunofluorescence and Confocal Microscopy

Cell surface labeling—Wild type and transfected MDCK cells were grown on fibronectin (BD Biosciences)-coated glass coverslips or in eight-well chambers (μ-Slide 8 Well, Ibidi, #80826) until day 6 post-confluence. Cells were rinsed with Ca/Mg buffer (phosphate-buffered saline containing 1 mM CaCl_2_ and 0.5 mM MgCl_2_) and incubated in blocking buffer (Ca/Mg buffer containing 0.2% gelatin) for 30 min at 4°C. Cells were incubated with either mouse mAb AC133 (epitope CD133/1; 1:50, Miltenyi Biotec GmbH, Bergisch Gladbach, Germany), AC141 (epitope CD133/2; 1:50, Miltenyi Biotec), 293C3 (CD133/2, 1:50, Miltenyi Biotec) or rat mAb 13A4 (1:100, eBioscience) diluted in blocking buffer for 1 h at 4°C. Cells were washed with PBS and then fixed in 4% paraformaldehyde (PFA) in PBS for 30 min at room temperature. Coverslips were then rinsed in PBS containing 50 mM NH_4_Cl and incubated in this same solution for 10 min.

Intracellular labeling—PFA-fixed cells were permeabilized and blocked with PBS containing 0.2% gelatin and 0.2% saponin (Panreac Applichem, Darmstadt, Germany) for 30 min at room temperature prior to the incubation with anti-prominin-1 antibodies for 30 min at room temperature. To visualize the primary cilium cells were labeled with a mouse mAb anti-acetylated α-tubulin (1:1000, clone 6-11B-1, #T6793, Sigma-Aldrich) followed by Alexa546-conjugated goat anti-mouse IgG_2b_ (1:600, Molecular Probes—Thermo Fisher Scientific) for 30 min in permeabilization buffer. For labeling with homemade antibodies, PFA-fixed cells were incubated in SDS buffer (0.005% SDS and 0.2% gelatin in PBS) for 30 min at room temperature and washed with PBS containing 0.2% gelatin for 10 min. Cells were labeled with mouse mAb 80B258 (10 μg/ml, [11]) or rabbit antiserum αhE2 (1:500, [10]) diluted in PBS containing 0.2% gelatin for 30 min at room temperature.

In all cases, Alexa555/546-conjugated goat/donkey anti-mouse, anti-rabbit or anti-rat IgG antibodies (1:500, Life Technologies) were used to detect anti-prominin-1 antibodies. Nuclei were counterstained with 4’-6-diamidino-2-phenylindole (DAPI, 1 μg/ml, Sigma-Aldrich). Samples were washed three times in PBS containing 0.2% gelatin, once in PBS, once in H_2_O and mounted in Mowiol 4.88 (Merck, Darmstadt, Germany). Cells were observed with a Zeiss LSM 700 confocal laser scanning microscope. The images in Fig 3A and S1 Video were prepared from data files using Volocity 3D Image Analysis Software (PerkinElmer) and all others were processed with Fiji [98].

### Flow Cytometry

Wild type and transfected MDCK cells were harvested via treatment with 0.05% trypsin/0.5 mM EDTA solution (Life Technologies) for 15 min at 37°C. Cells were centrifuged, and the pellet was resuspended in PBS containing 0.2% gelatin and 5 mM EDTA (VWR International, Radnor, Pennsylvania, USA). Cell suspensions (2 x 10^5^ cells in 100 μl) were incubated for 30 min at 4°C with fluorochrome-conjugated antibodies: mouse mAb AC133-phycoerythrin (PE) (1:10, Miltenyi Biotec), AC141-PE (1:10, Miltenyi Biotec), 293C3-allophycocyanin (APC) (1:10, Miltenyi Biotec) and CD34-PE or CD34-APC (1:10, BD Bioscience) or rat mAb 13A4-PE (1:10, eBioscience). Cells expressing prominin-1-GFP were examined using the fluorescein isothiocyanate (FITC) channel. Cells were washed with PBS containing 5 mM EDTA. After centrifugation, cells were resuspended and incubated for 10 min in 4% PFA. After washing with PBS containing 5 mM EDTA, 10,000 events were acquired on a FACSCanto II System (BD Bioscience). Instrument settings and gating strategies were established using unstained or CD34-labeled MDCK cells.

### Detergent Cell Extracts and Endoglycosidase Digestion

Wild type and transfected MDCK cells (2 x 10^6^) were grown in 6-well plates until 3 days post-confluence. Cells were harvested by trypsinization and centrifuged for 5 min at 300 x g. Cell pellets were lysed in solubilisation buffer (1% NP-40, 0.5% deoxycholate, 0.1% sodium dodecylsulfate, 150 mM NaCl, 50 mM Tris-HCl, pH 8.0) supplemented with complete protease inhibitor (Roche Molecular Biochemicals) for 30 min at 4°C. Lysates were centrifuged at 4°C (10 min, 10,000 x g) and the resulting supernatants were either used for endoglycosidase digestion or directly mixed with Laemmli sample buffer (with or without reducing agent; β-mercaptoethanol) prior to analysis with SDS-PAGE. For endoglycosidase digestion, the detergent cell extracts from MDCK cells expressing the canine prominin-1-GFP were incubated for 6 h at 37°C (or room temperature) in the absence or presence of 1 unit PNGase F (peptide-N-glycosidase F; #11 365 177 001, Roche Diagnostics GmbH).

### Immunoblotting

Cell detergent extracts were prepared from wild type or transfected MDCK cells (corresponding to one-tenth of a unit of 6-well plate) and separated by SDS-PAGE (7.5%) under reducing or non-reducing conditions and transferred to polyvinylidene difluoride (PVDF) membranes (pore size: 0.45 μm; Millipore Corp, Belford, MA) using a semi-dry transfer cell system (Cti, Idstein, Germany). After transfer, membranes were incubated in blocking buffer (PBS containing 0.3% Tween 20 and 5% milk powder) overnight at 4°C. Incubation with primary antibodies against prominin-1; mouse mAb AC133 (1:100), AC141 (1:100), 293C3 (1:100), 80B258 (1:1500) or rat mAb 13A4 (1:500), and rabbit antiserum αhE2 (1:1500) was performed for 1 h at room temperature. The GFP fusion protein was detected using a rabbit anti-GFP polyclonal antibody (1:2000, #210-PS-1GFP, ImmunoKontact). As a loading control, mouse mAb AC-74 against β-actin (1:10,000, Sigma-Aldrich) or DM1A against α-tubulin (1:10,000, Sigma-Aldrich) were used. All antibodies were diluted in blocking buffer. Antigen-antibody complexes were detected using appropriate horseradish peroxidase-conjugated secondary antibodies (1:5000, Jackson ImmunoResearch Inc.) and visualized with enhanced chemiluminescence reagents (ECL system, GE Healthcare). Membranes were exposed to films (GE Healthcare).

### Statistical Analysis

Statistical analysis was performed using unpaired two-tailed student’s t-test. Differences were regarded as not significant if the calculated *p*-value was ≥ 0.05.

## Supporting Information

S1 Fig Cloning strategy and 5’-untranslated regions of cDNA clones. (**A**) Schematic representation of the amplified cDNA clones obtained by PCR applying prominin-1-specific oligonucleotide primers (1 to 5, black triangles) alone or in combination with linker-specific adaptor primers (blue and green triangles) for the amplification of the 5’ and 3’ cDNA ends. The lines between the primers illustrate the amplified cDNA fragments and their size is indicated in bases (b). The red lines show the overlapping region between the PCR products. The names of the cDNA clones are indicated (see also below and S2 Fig). The clone CP4-A4 contains the facultative exon 3 (purple). The consensus cDNA sequences derived from the overlapping PCR products are shown as a blue boxed area for the coding region with single thick lines at the left and right representing the 5’- and 3’-untranslated regions (UTR), respectively. Dark blue zones highlight the positions of the five transmembrane domains (1–5). The A in parentheses indicates the presence of a poly-A tail. (**B**) Alternative exons in the 5’-UTR of canine prominin-1. Three 5’-UTR exons (A, B and C) were alternatively spliced prior to exon 1 (green), which encodes the initial codon. Their sequences are displayed as well as the names of the corresponding clones or the predicted ones (*X1* and *X2*) found in the database (GenBank entries are indicated in parentheses).
(TIF)

S2 Fig Facultative exon 22 and the C-terminal splicing cassette of canine prominin-1 are conserved in mouse, rat and human. (**A**) Exon 22 (brown) at the end of the second extracellular loop of canine prominin-1 (GenBank Accession No. KR758755) is skipped in one clone amplified by 3’-RACE resulting in the in-frame deletion of 23 residues as indicated. (**B**) Identification of exon 26a. Exons 24 and 27 from the cDNA clones (top) are displayed in green and blue, respectively, and exon 26a from the genomic clone (Accession No. NW_003726054.1) in red (bottom). The corresponding deduced amino acid sequences are indicated above. The donor and acceptor splice sites (GT/AG) are indicated in boxes and the polyadenylation signal (AAUAAAA) is underlined. The poly-A tail is shown. (**C**) The alternative exon 26a is conserved among mammalian species. The amino acid sequences deduced from dog, rat and human genomes are shown in italics whereas the sequence translated from murine mRNA appears in regular font (red) [21]. Arrowhead, exon boundary.
(TIF)

S3 Fig The GFP tag did not impede the immunodetection of prominin-1-GFP fusion protein. (**A**) Stably transfected MDCK cells expressing human prominin-1-GFP (green) were cell surface-immunolabeled with AC133, AC141, 293C3 and 13A4 mAbs followed by the appropriate Alexa555-conjugated secondary antibody (red). As a negative control, only the secondary antibody was used as indicated. The insets show an enlargement of the apical plasma membrane where a double staining is observed, which is typical for microvilli (arrowhead). (**B, C**) Detergent lysates prepared from MDCK cells stably transfected with human or canine prominin-1-GFP as well as wild type cells (MDCK) were analyzed by SDS-PAGE under reducing (B) and non-reducing (C) conditions and immunoblotting. Antibodies are indicated. β-actin and α-tubulin were used as loading controls. GFP and prominin-1 immunoreactivities are indicated (green and black bracket, respectively). Asterisks show potential prominin-1 dimers or multimers. Filled circle, human prominin-1 degradation fragments. Molecular mass markers (kDa) are indicated. The original and uncropped blots are presented in S5 Fig. Note that anti-human prominin-1 antibodies recognize the human prominin-1-GFP fusion protein while the anti-mouse prominin-1 antibody (13A4) does not. (**D**) The expression levels of human and canine prominin-1-GFP were quantified upon normalization to the α-tubulin expression. They were not significant (ns) different as revealed by two-tailed unpaired student’s t-test (n = 3). Scale bar, 15 μm.
(TIF)

S4 Fig Instrument setup and flow cytometry controls. (**A, B**) MDCK cells were incubated without (A) or with anti-human CD34 antibody conjugated either to PE or APC fluorochrome (B) and analyzed by flow cytometry using the appropriate channel. The FITC channel was used to detect GFP. Unstained cells and those labeled with the irrelevant fluorochrome-conjugated antibody were used to set up the cut-off for positive cells.
(TIF)

S5 Fig Uncropped scans of blots shown in Figs 5 and 6 and S3 Fig. The information presented in Figs 5 and 6 and S3 Fig are indicated in red boxes. The molecular weight markers are presented. The black line illustrates membrane cutting.
(TIF)

S1 Table Exon-intron boundaries of the canine *prom1* gene. (DOCX)

S2 Table Oligonucleotide primers used in this study. (DOCX)

S1 Video Localization of canine prominin-1-GFP in microvilli and the primary cilium of MDCK cells. Confluent MDCK cells expressing canine prominin-1-GFP (green) were fixed, permeabilized and immunolabeled with mAb against acetylated α-tubulin (red) to reveal the primary cilium. Their nuclei were counterstained with DAPI (blue). Image was acquired by confocal laser scanning microscope and processed using Volocity software.
(MOV)
