# Supplementary figures and images for: Monoclonal Antibodies 13A4 and AC133 Do Not Recognize the Canine Ortholog of Mouse and Human Stem Cell Antigen Prominin-1 (CD133)
Source: PLoS One. 2016 Oct 4;11(10):e0164079. doi: 10.1371/journal.pone.0164079 (PMC5049760; doi:10.1371/journal.pone.0164079)

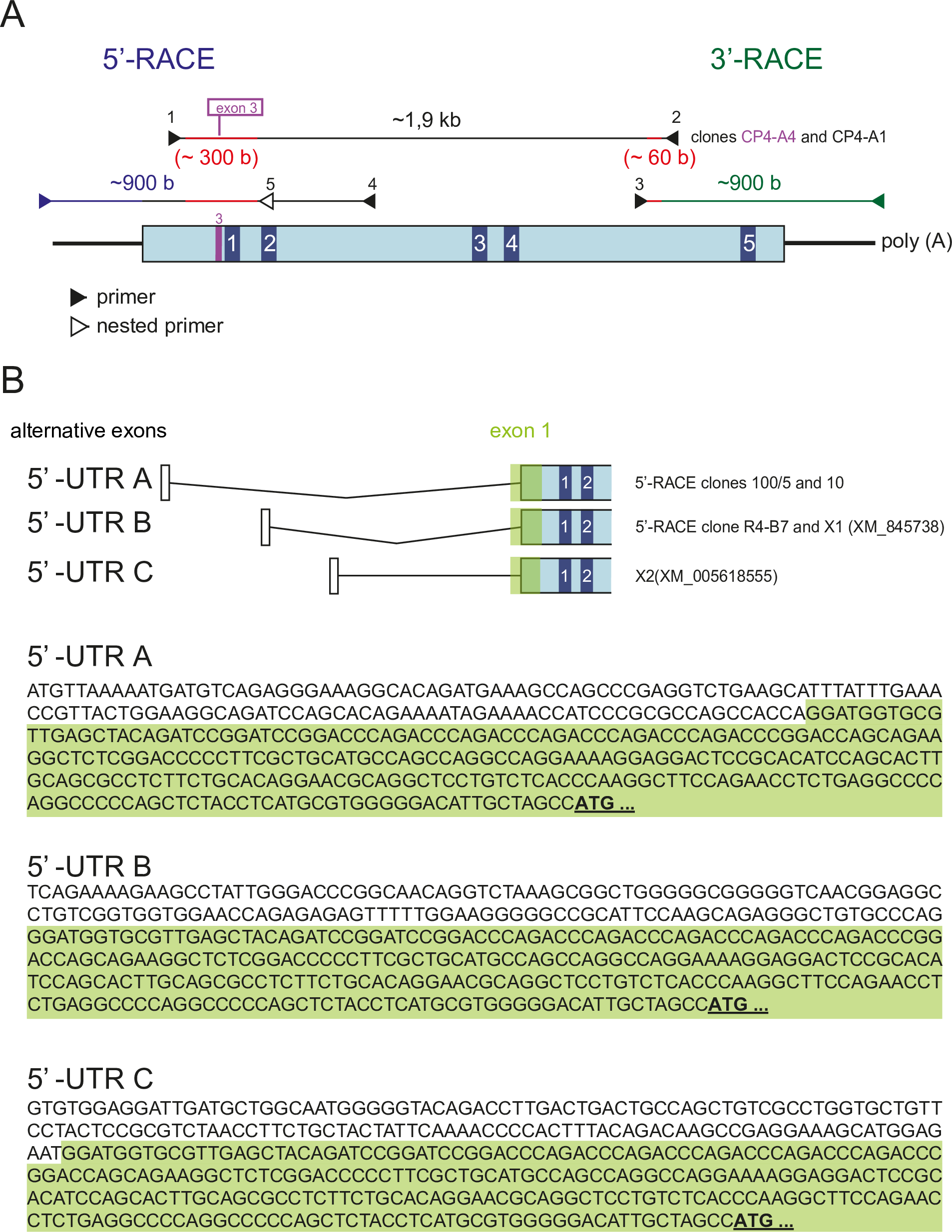

Supplement: S1 Fig — (A) Schematic representation of the amplified cDNA clones obtained by PCR applying prominin-1-specific oligonucleotide primers (1 to 5, black triangles) alone or in combination with linker-specific adaptor primers (blue and green triangles) for the amplification of the 5’ and 3’ cDNA ends. The lines between the primers illustrate the amplified cDNA fragments and their size is indicated in bases (b). The red lines show the overlapping region between the PCR products. The names of the cDNA clones are indicated (see also below and S2 Fig). The clone CP4-A4 contains the facultative exon 3 (purple). The consensus cDNA sequences derived from the overlapping PCR products are shown as a blue boxed area for the coding region with single thick lines at the left and right representing the 5’- and 3’-untranslated regions (UTR), respectively. Dark blue zones highlight the positions of the five transmembrane domains (1–5). The A in parentheses indicates the presence of a poly-A tail. (B) Alternative exons in the 5’-UTR of canine prominin-1. Three 5’-UTR exons (A, B and C) were alternatively spliced prior to exon 1 (green), which encodes the initial codon. Their sequences are displayed as well as the names of the corresponding clones or the predicted ones (X1 and X2) found in the database (GenBank entries are indicated in parentheses). (TIF) [file pone.0164079.s001.tif]

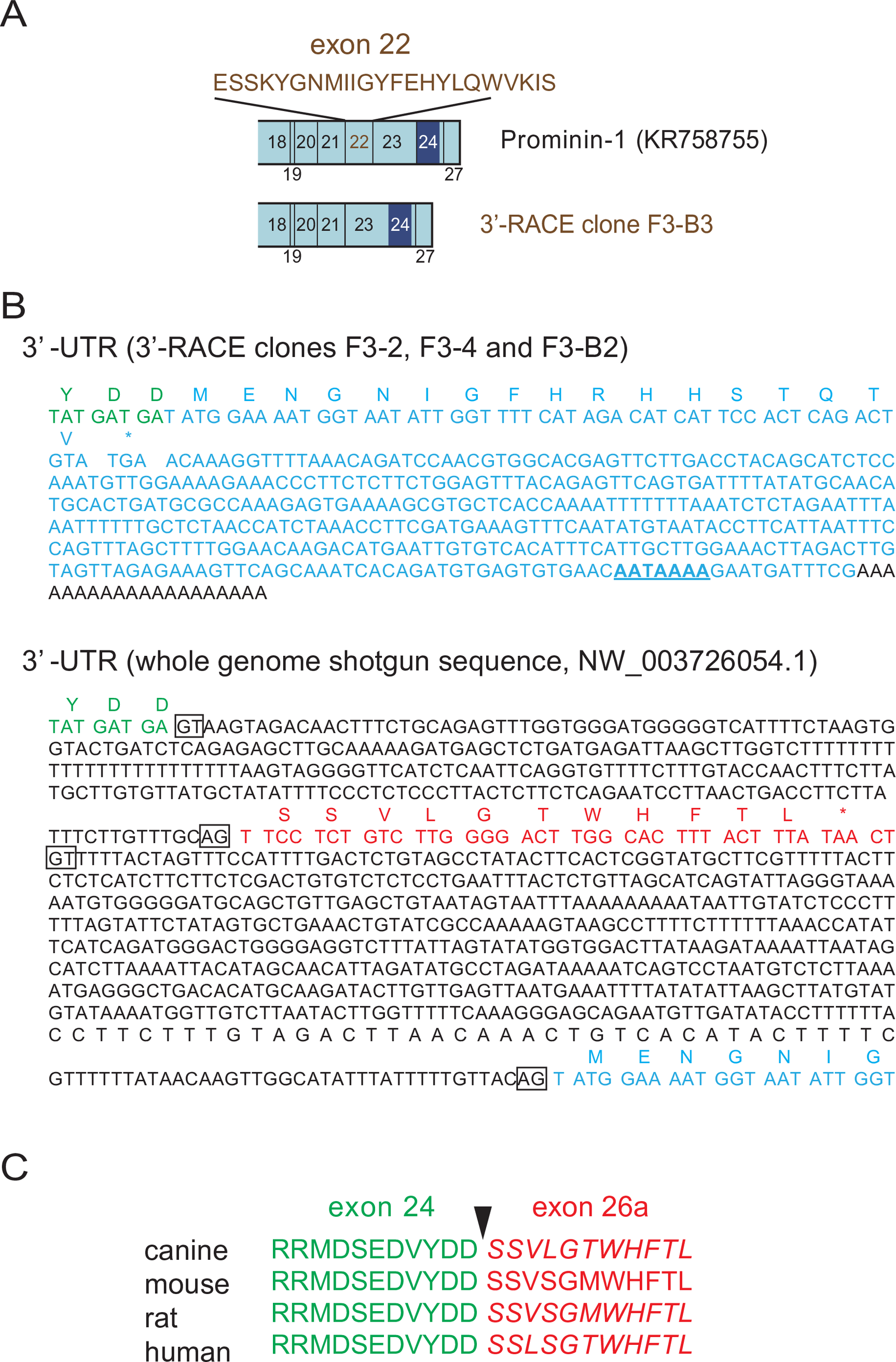

Supplement: S2 Fig — (A) Exon 22 (brown) at the end of the second extracellular loop of canine prominin-1 (GenBank Accession No. KR758755) is skipped in one clone amplified by 3’-RACE resulting in the in-frame deletion of 23 residues as indicated. (B) Identification of exon 26a. Exons 24 and 27 from the cDNA clones (top) are displayed in green and blue, respectively, and exon 26a from the genomic clone (Accession No. NW_003726054.1) in red (bottom). The corresponding deduced amino acid sequences are indicated above. The donor and acceptor splice sites (GT/AG) are indicated in boxes and the polyadenylation signal (AAUAAAA) is underlined. The poly-A tail is shown. (C) The alternative exon 26a is conserved among mammalian species. The amino acid sequences deduced from dog, rat and human genomes are shown in italics whereas the sequence translated from murine mRNA appears in regular font (red) [21]. Arrowhead, exon boundary. (TIF) [file pone.0164079.s002.tif]

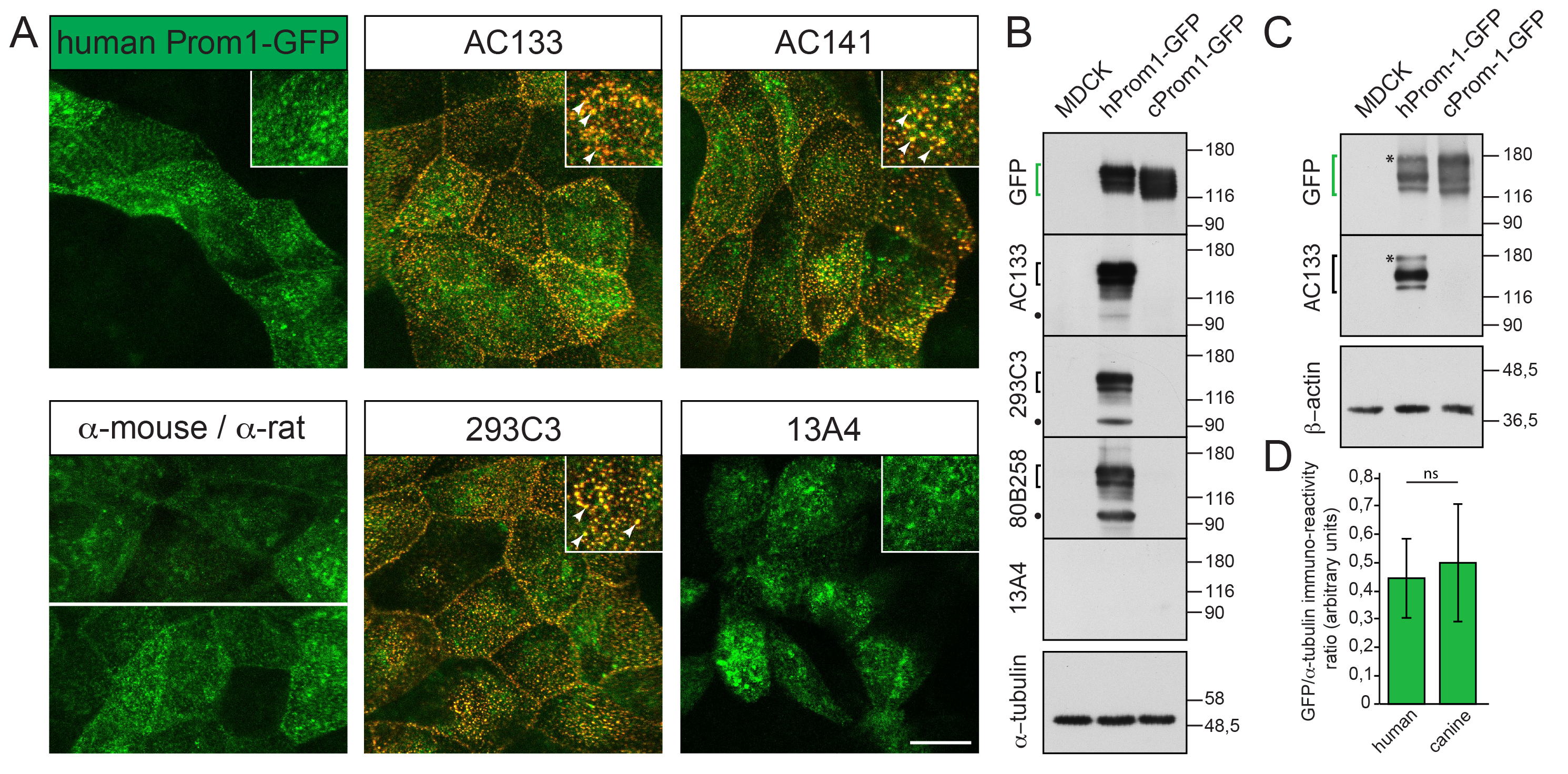

Supplement: S3 Fig — (A) Stably transfected MDCK cells expressing human prominin-1-GFP (green) were cell surface-immunolabeled with AC133, AC141, 293C3 and 13A4 mAbs followed by the appropriate Alexa555-conjugated secondary antibody (red). As a negative control, only the secondary antibody was used as indicated. The insets show an enlargement of the apical plasma membrane where a double staining is observed, which is typical for microvilli (arrowhead). (B, C) Detergent lysates prepared from MDCK cells stably transfected with human or canine prominin-1-GFP as well as wild type cells (MDCK) were analyzed by SDS-PAGE under reducing (B) and non-reducing (C) conditions and immunoblotting. Antibodies are indicated. β-actin and α-tubulin were used as loading controls. GFP and prominin-1 immunoreactivities are indicated (green and black bracket, respectively). Asterisks show potential prominin-1 dimers or multimers. Filled circle, human prominin-1 degradation fragments. Molecular mass markers (kDa) are indicated. The original and uncropped blots are presented in S5 Fig. Note that anti-human prominin-1 antibodies recognize the human prominin-1-GFP fusion protein while the anti-mouse prominin-1 antibody (13A4) does not. (D) The expression levels of human and canine prominin-1-GFP were quantified upon normalization to the α-tubulin expression. They were not significant (ns) different as revealed by two-tailed unpaired student’s t-test (n = 3). Scale bar, 15 μm. (TIF) [file pone.0164079.s003.tif]

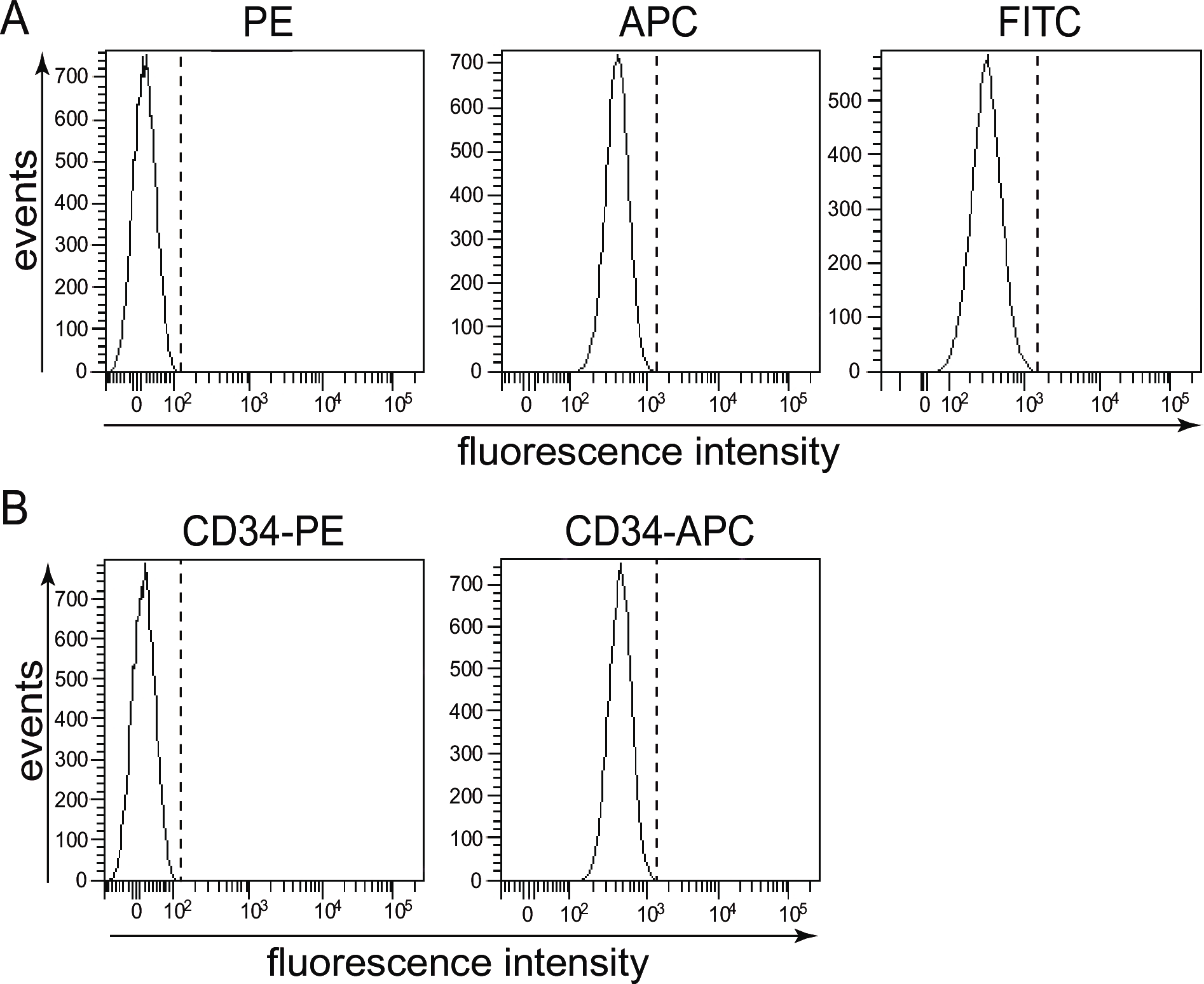

Supplement: S4 Fig — (A, B) MDCK cells were incubated without (A) or with anti-human CD34 antibody conjugated either to PE or APC fluorochrome (B) and analyzed by flow cytometry using the appropriate channel. The FITC channel was used to detect GFP. Unstained cells and those labeled with the irrelevant fluorochrome-conjugated antibody were used to set up the cut-off for positive cells. (TIF) [file pone.0164079.s004.tif]

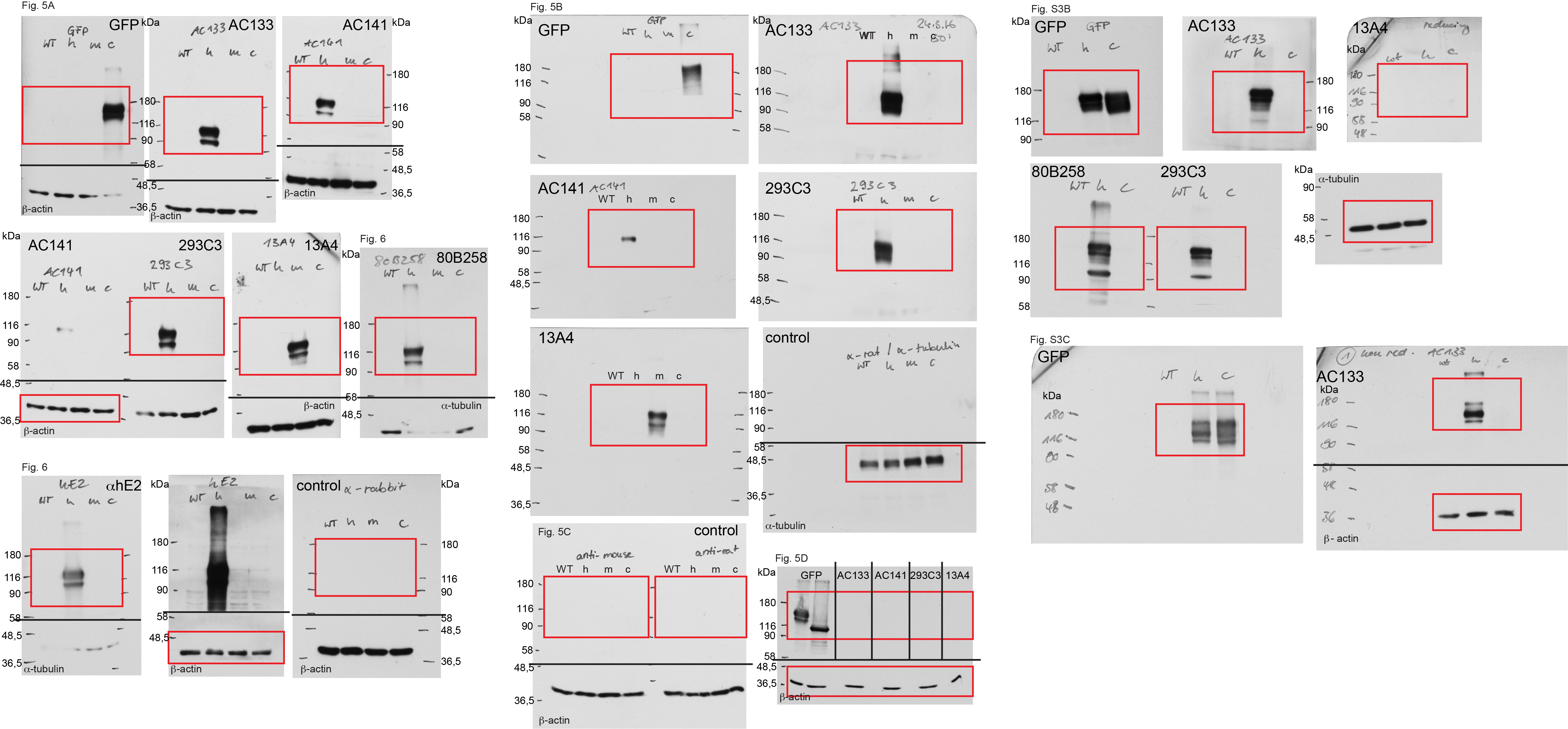

Supplement: S5 Fig — The information presented in Figs 5 and 6 and S3 Fig are indicated in red boxes. The molecular weight markers are presented. The black line illustrates membrane cutting. (TIF) [file pone.0164079.s005.tif]
