# Supplementary material for: Monoclonal Antibodies 13A4 and AC133 Do Not Recognize the Canine Ortholog of Mouse and Human Stem Cell Antigen Prominin-1 (CD133)
Source: PLoS One. 2016 Oct 4;11(10):e0164079. doi: 10.1371/journal.pone.0164079 (PMC5049760; doi:10.1371/journal.pone.0164079)
Supplement: S1 Table — (DOCX) [file pone.0164079.s006.docx]

| Exon No. | 3’ splice site | 5’ splice site | nt in cDNA  (XM_845738) |
| --- | --- | --- | --- |
| 5’-UTR A  (clones 100/5 and 10) | AAATT | CACCAgtaag | *1-159* |
| 5’-UTR C  (XM_005618555)  5’-UTR B  (clone R4-B7 and XM_845738)  1  2  3*  4  5  6  7  8  9  10  11a*§  11  12  13  14  15  16  17  18  19*  20  21  22  23  24  [25]  {26a}  [26b]  27 | GTGTG  TCAGA  accagGGATG  cccagATATT  cacagCCAGA  tgcagATTAT  tgcagTGTGG  tttagCAAAT  tttagATATT  tacagCAATC  ttcagCTCCC  tctagGGCTA  tgcagCCTCC  ttcagATGTC  accagGTGGC  tccagCGGAG  tctagATTTT  cacagTGACT  tgcagCATGC  tgtagATGGG  tgcagCCCCA  ccaagAAATA  accagAGCAC  tccagGTGAA  tccagGAAAG  catagATTAC  cctagAATTT  –  tgcagTTCCT  –  tacagTATGG | AGAATgtaag  CCCAGgtgag  CGAAGgtaag  ATAAGgtaat  TGAAGgtaaa  ATAAGgcaag  CAGCGgtaaa  GGATAgtgag  TGAAGgtaag  GTCAGgtgag  AAAAGgtatg  ATCAGgtgag  CCCTgtaag  TACAGgtgag  ATGGTgtgag  TCCAGgttaa  TACAGgtata  AGGAGgtaag  CAGAGgtgtg  ATCTGgtgag  CAATGgtaac  CGAGGgtaag  TGAGGgtaag  TCAAAgtaag  TCTCAgtaag  CTATGgtaag  GATGAgtaag  –  TAACTgtttt  –  TCTGA | *1-144*  1-136  137-615  616-671  672-698  699-904  905-1025  1026-1089  1090-1179  1180-1397  1398-1472  1473-1536  1537-1545  1546-1705  1706-1859  1860-1982  1983-2086  2087-2171  2172-2318  2319-2390  2391-2482  2483-2501  2501-2555  2556-2636  2637-2705  2706-2798  2799-2914  –  –  –  2915-3351 |

**S1 Table.** Exon-intron boundaries of the canine *prom1* gene.

The exon-intron boundaries were determined by comparing the canine prominin-1 cDNA sequences (GenBank Accession Nos. KJ654317, KR758755, XM_005618555, and XM_845738) with canine whole genome shotgun sequence (Accession No. NW_003726054.1). The 5’-UTR A, B and C refer to potential alternative exons within the 5’-unstranslated regions of canine prominin-1 cDNA clones as indicated in brackets, while exons numbered 1 to 27 concern the open reading frame. The nucleotide (nt) position is annotated according to the XM_845738 sequence except for 5’-UTR A and C, which refer to our isolated cDNA clones (100/5 and 10) and XM_00561855 sequences, respectively. The 5’ and 3’ ends of each exon appear as uppercase characters with the adjacent intron 3’ acceptor site and 5’ donor site as lowercase letters.

*Facultative exons.

§Mini-exon predicted in canine prominin-1 but not reported yet in other mammalian prominin-1 [20].

The exons in brackets are absent in canine transcripts by comparison to the human and/or mouse prominin-1 sequences while the exon in braces is predicted from the genomic sequence (S2B Fig) [1].
