# Supplementary material for: Monoclonal Antibodies 13A4 and AC133 Do Not Recognize the Canine Ortholog of Mouse and Human Stem Cell Antigen Prominin-1 (CD133)
Source: PLoS One. 2016 Oct 4;11(10):e0164079. doi: 10.1371/journal.pone.0164079 (PMC5049760; doi:10.1371/journal.pone.0164079)
Supplement: S2 Table — (DOCX) [file pone.0164079.s007.docx]

**S2 Table.** Oligonucleotide primers used in this study.

| No. | Name | | Nucleotide (nt) sequence | nt position in cDNA§ | |
| --- | --- | --- | --- | --- | --- |
| **cDNA amplification** | | | | | |
| 1 | MDCK-P1_6 fw | | 5’-TGTTCCTCCAAGTGGTGCAG-3’ | 247-266 | |
| 2 | MDCK-P1_6 rv | | 5’-TGGTTGATAGTGCTCATTGACT-3’ | 2158-2137 | |
| 3 | RACE-3 fw | | 5’-CACCATGGCCAAGTCATGCCTTTGG-3’ | 2109-2133 | |
| 4 | RACE-1 rv | | 5’-TAGCCACTGGAGGGACAGAGCAC-3’ | 1005-983 | |
| 5 | RACE-4 rv (nested) | | 5’-CGATCCGGGTCCTCAGGTGGTG-3’ | 627-606 | |
| 6 | MDCKP1_2.1F | | 5’-CCCAAGGCTTCCAGAACCTCTGAG-3’ | (-6)-18 | |
| 7 | MDCKP1_4R | | 5’-AGAACTCGTGCCACGTTGGATCTG-3’ | 2626-(2650) | |
|  | | | | | |
| **Subcloning#** | | | | | |
| 8 | 5’UTR-XhoI | | 5’-CTCCTGTCTCA**CTCGAG**GCTTCCAG-3’ | (-17)-8 | |
| 9 | 5’UTR-SbfI-BamHI | | 5’-ATGG**GGATCC**ACCA**CCTGCAGG**AACA-3’ | 247-272 | |
| 10 | CP-SbfI | | 5’-GCAAGTGTT**CCTGCAGG**TGGTGCAGCCCC-3’ | 242-270 | |
| 11 | CP-SacI-BamHI | | 5’-CCT**GGATCC**TTTGGTTGATA**GAGCTC**ATTGAC-3’ | 2138-2169 | |
| 12 | 3’UTR-SacI | | 5’-GTCAAT**GAGCTC**TATCAACCAAAG-3’ | 2138-2161 | |
| 13 | 3’UTR-BamHI | | 5’-GTGCCACGTT**GGATCC**GTTTAAAACC-3’ | 2618-(2643) | |
| 14 | 3’del new 2.1 fw | | 5’-CCACTCAGACTGTAACAAAGGTTTTAACGGATCCACC-3’ | 2596-2636 | |
| 15 | 3’del new 2.1 rv | | 5’-GGTGGATCCGTTAAAACCTTTGTTACAGTCTGAGTGG-3’ | 2636-2596 | |
|  | | | | | |
| **Deletion of created restriction sites for subcloning** | | | | | |
| 16 | SbfI-remove fw | 5’-GTGCAAGTGTTCCTCCAAGTGGTGCAGCCCCA-3’ | | | 240-271 |
| 17 | SbfI-remove rv | 5’-TGGGGCTGCACCACTTGGAGGAACACTTGCAC-3’ | | | 271-240 |
| 18 | SacI-remove fw | 5’-CTTTGGAACAGTCAATGAGCACTATCAACCAAAGTATCAAG-3’ | | | 2128-2168 |
| 19 | SacI-remove rv | 5’-CTTGATACTTTGGTTGATAGTGCTCATTGACTGTTCCAAAG-3’ | | | 2168-2128 |

§ Nucleotide position refers to the canine prominin-1 sequence deposited in database under the accession number KR758755.

# The introduced enzyme restriction sites are shown in bold letters.

Numbers in parentheses refer to prominin-1 sequence absent in the sequence KR758755, but appears in 5'-UTR (S1B Fig) or 3'-UTR (S2B Fig) sequences.

Fw, forward primer; rv, reverse primer.
